# Supplementary material for: Selection, Identification and Functional Performance of Ammonia-Degrading Microbial Communities from an Activated Sludge for Landfill Leachate Treatment
Source: Microorganisms. 2023 Jan 25;11(2):311. doi: 10.3390/microorganisms11020311 (PMC9961800; doi:10.3390/microorganisms11020311)
Supplement: Supplementary file 1 [file microorganisms-11-00311-s001.zip › microorganisms-2144116-supplementary.pdf]

Parameters	Leachate (A)	Leachate (B)	Leachate (C)	Leachate (D)
Color	Dark brown	Dark brown	Dark brown	Dark brown
Smell	Strong	Strong	Strong	Very Strong
pH	8,28	8,40	6,00	8,89
NH_4^+-N (mg/L)	4427	2200	1140	1560
COD (mg/L)	5640	5000	2600	117000
BOD (mg/L)	640	1500	910	50370
BOD/COD	0,11	0,30	0,35	0,43
Cl^-	1806	3263	1430	3844

Supplementary Table S1.**Composition of different leachates.** The characteristics of four different leachates, such as (A) active landfill only special waste, (B) active landfill with pre-treated municipal waste, (C) post operational phase landfill and (D) landfill with high organic content waste, are shown. The nitrogen content in leachates is present almost only as ammoniacal nitrogen (NH_4^+-N).

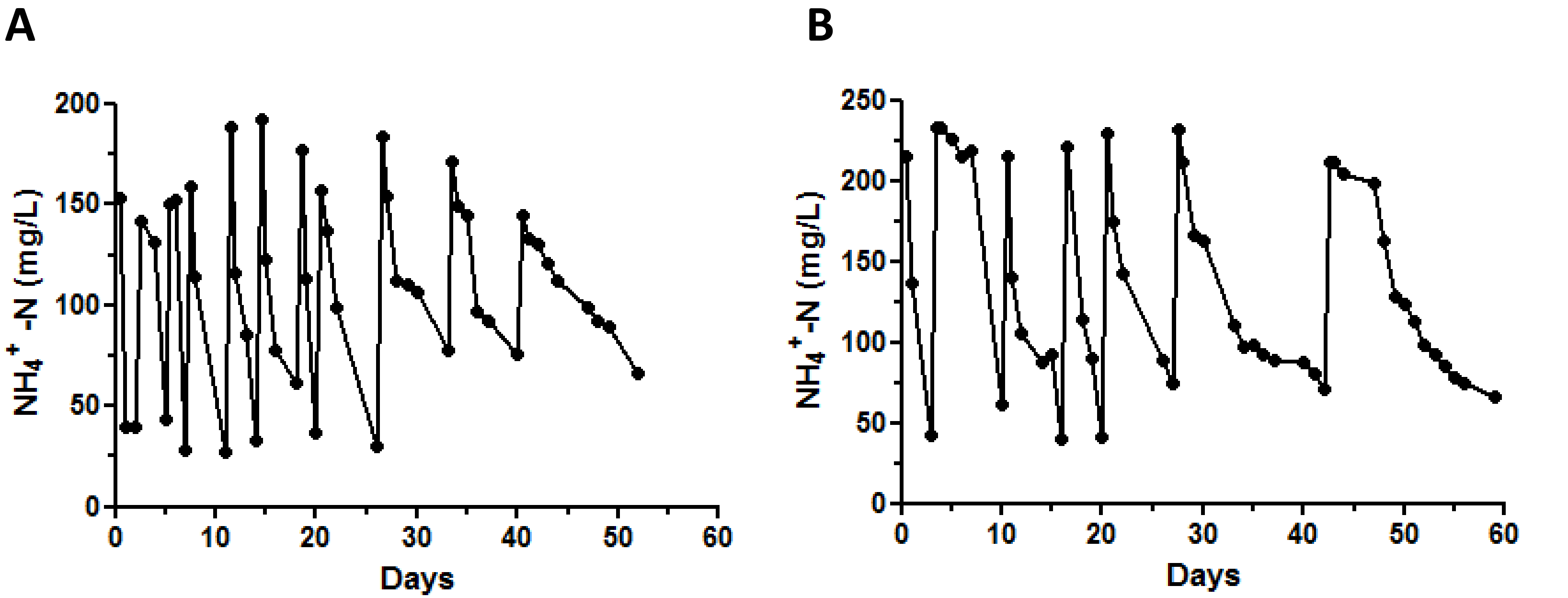

Supplementary Figure S1. Repetitive Re-Inoculum Assay. RRIAs were carried out essentially as described in the text and in the legend of Figure 3. At the starting point (day 0), the ammonia concentrations, expressed as NH_4^+-N, were 150 (**A**) and 220 (**B**) mg/L . At each NH_4^+ fall, an aliquot of the culture was re-inoculated in fresh LMM medium and ammonia concentration was adjusted over again to the initial value (peaks). The two cultures subjected to RRIA were followed for about two months days.

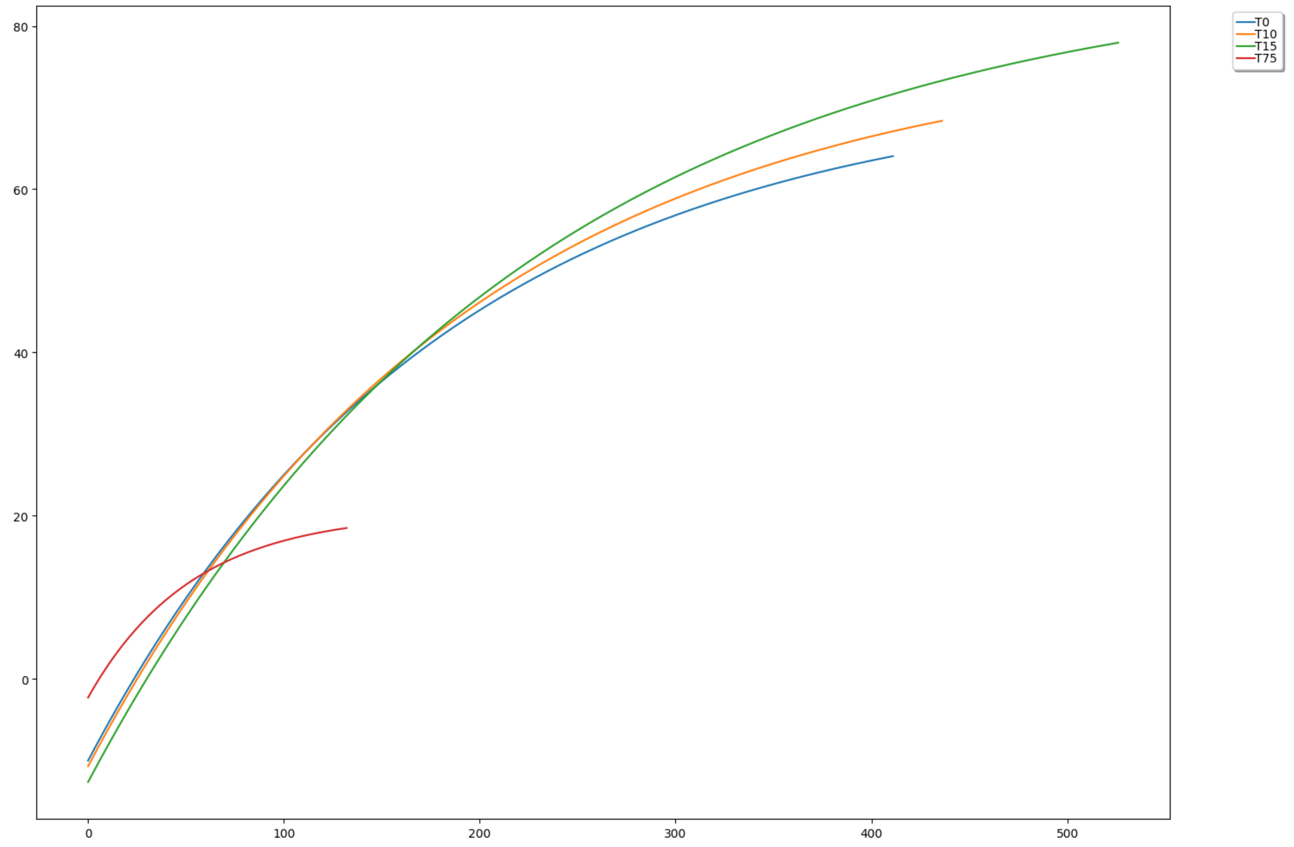

Supplementary Figure S2. Rarefaction curves obtained from 16S rRNA amplicon NGS samples collected at time point 0, 10, 15 and 75. The number of different OTUs on the y axis is plotted vs. the number of sequences on the x axis.
